# Seasonal dynamic of cholecalciferol (D3) and anti-Muellerian hormone (AMH) with impact on ovarian response and IVF/ICSI

**DOI:** 10.1007/s00404-022-06419-1

**Published:** 2022-02-27

**Authors:** Nina Rogenhofer, Udo Jeschke, Viktoria von Schönfeldt, Sven Mahner, Christian J. Thaler

**Affiliations:** 1 Division of Gynecological Endocrinology and Reproductive Medicine, Department of Obstetrics and Gynecology, University Hospital LMU Munich, Marchioninistrasse 15, 81377 Munich, Germany; 2 Department of Obstetrics and Gynecology, University Hospital Augsburg, Stenglinstrasse 2, 86156 Augsburg, Germany

**Keywords:** Vitamin D, Cholecalciferol, D3, AMH, Assisted reproductive technologies, IVF, ICSI

## Abstract

**Objective:**

Recent studies revealed intriguing associations between cholecalciferol (D3) and reproductive functions. Seasonal changes of D3 concentrations are well known; however, they are not always considered in the context of reproductive functions. In this study, we analyzed D3 serum concentration in IVF/ICSI patients with respect to seasonal 3-month quartiles and anti-Muellerian hormone (AMH) referring to the impact on Assisted Reproductive Technologies (ART) outcome.

**Materials and research methods:**

We studied 469 female patients, presenting between 2012 and 2018 for ART treatment in our fertility center. D3 as well as the AMH serum concentrations were measured at the beginning of the follicle stimulation (days 3–5 of menstrual cycles). Results were evaluated with respect to seasonal quartiles and outcome of the ART cycles.

**Results:**

D3 concentrations showed significant fluctuations within annual quartiles with a pronounced peak in August–October and a minimum in February–April (26.0 vs. 20.5 mg/dl; *p* < 0.0001). Similar seasonal dynamics were found for AMH (2.98 vs. 1.78 ng/ml; *p* = 0.010) and these were associated with significantly shorter stimulation periods during August–October (11.29 vs. 12.12 days; *p* = 0.042), higher number of fertilized oocytes between August and October (6.23 vs. 4.97; *p* = 0.05) along with a trend towards higher numbers of cumulus–oocyte complexes. However, no such differences were found for the numbers of MII oocytes or pregnancy rates.

**Conclusion:**

Our data indicate seasonal 3-month quartile variations of AMH concentrations and characteristics of ART, such as days of ovarian stimulation and number of fertilized oocytes. Highest AMH concentrations were found between August and October and this quartile was associated with highest D3 concentrations.

## Introduction

Vitamin D is a steroid hormone, mainly produced by the skin upon exposure to sunlight, with less than 20% provided by alimentary sources [1]. It is hepatically converted to 25-hydroxyvitamin D (25OH-D) and transformed in the kidneys by renal 1a-hydroxylase into the biologically active form 1,25-dihydroxy-vitamin-D (1,25(OH)2-D3), i.e., calcitriol/cholecalciferol [2]. Although 25(OH)-D has some metabolic activity, 1,25(OH)2-D3 (D3) is the most active metabolite of vitamin D [3]. Accumulating data indicate the influence of D3 on different aspects of human health, fertility and reproductive functions in particular [1, 2, 4–9]. In fact, vitamin D receptors (VDR) have been identified not only in calcium-regulating tissues such as the intestines, skeleton and parathyroid glands, but also in reproductive organs, such as ovaries, uterus, placenta, granulosa cells [10], testis and hypothalamus [3, 10, 11]. A recent study described that D3 deficiency was associated with polycystic ovary syndrome (PCOS) [12] and D3 supplementation improved menstrual problems, dysmenorrhea and premenstrual syndrome [13]. In addition, D3 stimulated estrogene and progesterone production in the human placenta [1, 14] and D3 was reported to play a role in regulating the thickness of the endometrium and the number of antral follicles in the ovaries, indicating a beneficial effect of vitamin D on female fertility [15].

One of most important biomarkers, which plays an important role in folliculogenesis, is the anti-Muellerian hormone (AMH). AMH is a member of the transforming growth factor-beta superfamily produced by the granulosa cells [16]. The relatively small fluctuations of serum AMH during the menstrual cycle, the strong correlation with the primordial oocyte pool size as well as the follicular response to ovarian stimulation, makes it superior to other ovarian reserve markers (such as day 3 FSH and inhibin), clinically useful and convenient for patients [1, 17]. AMH is influenced by a wide spectrum of endogenous and exogenous factors and these may account for a wide variation of AMH even for women of comparable age [1, 18]. Current data point to the D3 status as one additional factor affecting AMH concentrations, since early in vitro studies detected a vitamin D response element in the promotor of the AMH gene, providing a direct molecular link for vitamin D effects on AMH gene expression [19]. In accordance, data demonstrated that D3 could increase AMH production via activating the vitamin D response element in the AMH gene [19, 20]. Resulting data suggested that D3 affects ovarian reserve along with the ability to modify AMH production [2, 21]. Based on accumulating evidence that D3 supplementation may be beneficial for fertility and pregnancy, it was approved that seasonal changes in AMH concentrations can be annihilated by appropriate supplementation of D3 in vitamin D-depleted women [2, 18, 22].

A recent review and meta-analysis evaluating the relationship between serum vitamin D and AMH levels report quite discrepant results [1]. Several studies claimed a positive correlation between serum vitamin D and AMH [1, 18, 23–25], while others postulate negative correlations [1, 4, 21, 26–35]. However, only a few of the numerous studies respect the seasonal fluctuations of vitamin D even though this phenomenon is well known.

So far, there are only limited and mainly contradicting publications concerning the seasonal fluctuations of vitamin D and the impact to AMH, respectively, the relation to fertility [1].

Up to now, there are no data analyzing seasonal 3-month changes of D3 with regard to AMH and ART (Assisted Reproductive Technologies), i.e., in vitro fertilization (IVF), intracytoplasmatic sperm injection (ICSI) outcome. Just since we reported in our pilot study 3-month fluctuations of D3 serum concentrations with delayed peak in autumn (August to end of October) postulating a prolonged tissue storage with a following slow release of D3 resulting in a delay of the D3 deficiency [36].

Therefore, we analyzed D3 serum concentrations in IVF and ICSI patients with respect to seasonal 3-month quartiles and AMH referring to the impact on ART outcome.

## Materials and methods

### Patients

D3 serum concentrations and AMH were analyzed in patients undergoing ART in our fertility Center between December 2012 and August 2018. During this period, 4078 patients presented for ART, 576 patients agreed to take part in this study and 469 of them met the study criteria and signed informed consent. Further study criteria are summarized in Table 1.

**Table 1:** Study criteria

Study criteria	
Signed informed consent	
Age ≥ 20 ≤ 43 years	
European origin	
No intake of Vitamin D supplements	
No women with iatrogenic (e.g., gonadotoxic therapy, surgery) or known genetic cause of ovarian insufficiency	
Non smoker	
No intake of medications for systematic diseases	e.g., anti-epileptics, anti-depressives
No intake of corticosteroids	
Exclusion of severe or chronic diseases	Gastric, renal, hepatic, cardiac, skeleton
Exclusion of infectious diseases	Hepatitis, HIV, Tuberculosis
Heterologous partners	

Indications for IVF or ICSI were female factors such as blocked fallopian tubes (16.8%, *n* = 79), endometriosis (7.3%, *n* = 34), male factors such as low sperm count, oligo-astheno-teratozoospermia, sperm antibodies (46.1%, *n* = 216) or idiopathic (29.8%, *n* = 140). Data are illustrated in Table 2.

**Table 2:** Indications for assisted reproductive technologies (ART)

	Number (*n*) %
Male factors	
Low sperm count, oligo-astheno-teratozoospermia (oat), sperm antibodies	216 46.1
Female factors	
Blocked fallopian tubes	79 16.8
Endometriosis	34 7.3
Idiopathic infertility	140 29.8

Idiopathic no reason detectable for conceiving after trying to get pregnant for 1 year or more

All included patients were of European descent and living in Europe, mainly in Germany, hence being exposed approximately to identical hours of sunlight per day. The participants were healthy women without severe diseases who failed to conceive more than 1 year. They did not take any vitamin D supplementation, otherwise these patients were excluded. Also, women with polycystic ovary syndrome (PCOS, according to Rotterdam criteria [37]) were ruled out, because these patients are known to reveal increased AMH levels [1, 12]. Patients with known genetic or iatrogenic (e.g., gonadotoxic therapy, surgery) causes for premature ovarian failure were ineligible, as well. (Table 1).

### Informed consent and ethical approval

Blood was only drawn with signed informed consent. The present study complied with the ethical guidelines of the institution. All procedures performed in this study involving human participants were in accordance with the ethical standards and the national research committee and with the 1964 Helsinki declaration and the later amendments or comparable ethical standards. The present study complied with the ethical guidelines of the institution. The Human Investigation Review Board of the Ludwig-Maximilians-University Munich approved the study (IRB No 671-15).

### Analysis

Biographic data and measurements of D3 serum concentrations, AMH, AFC (antral follicle count) and BMI (Body Mass Index) were recorded on the same day as we started controlled hyperstimulation for IVF/ICSI treatment, respectively, between day 3 and day 5 of bleeding (Table 3). AFC was performed by a transvaginal sonography representing the total antral follicles of both ovaries measuring between 2 and 10 mm.

**Table 3:** Biographic data of the patients (*n* = 469) on day of beginning IVF/ICSI program, respectively, day of starting follicle stimulation

	Mean ± STD (range)
Age years	34.8 ± 3.4 21–43
BMI kg/m^2^	20.7 ± 2.1 19–35
AFC	8.4 ± 5.1 1–19
AMH ng/ml	2.51 ± 2.50 0.02–17
Vitamin D3 ng/ml	23.51 ± 10.89 14–73.4

*BMI* body mass index (Calculated as weight in kilograms divided by the square of height in meters), *AFC* antral follicle count, *AMH* Anti-Muellerian Hormone

### Biochemical analysis

Vitamin D levels were analyzed in patient sera with the quantitative DiaSorin Liaison. Chemiluminescence Immunoassay (CLIA) on the Liaison & Liaison XL analyzer (DiaSorin Austria, Vienna, Austria), as per the manufacturer guidelines. The system detects 1,25(OH)2-D3 with a range of 4–150 ng/ml with no significant cross reactivities of other metabolites. The limit of detection was 4 ng/ml with 95% probability of detection. The intra- and inter-assay coefficients of variations were 10 and 15%, respectively.

Serum AMH levels were assessed with the fully automated Electrochemiluminescence immunoassay (ECLIA) for quantitative determination of AMH in human serum and plasma (Elecsys Cobas, Roche Diagnostics International Ltd., Rotkreuz, Switzerland). The Elecsys Cobas AMH was analyzed on a Cobas 6000 e601 platform using Roche’s standard protocol.

The AMH immunoassay demonstrated a within-run-precision coefficients of variation (CV) of below 3.4%. The repeatability precision does not exceed 1.8%. The limit of quantitation (LOQ) was 0.07 ng/ml.

### ART procedure

Controlled hyperstimulation was performed with following protocols: the GnRH (gonadotropin releasing hormone) agonist protocol starting with administration of GnRH agonist (e.g., Nafarelin 0.8 mg/d nasal) on cycle-day 23 followed by administration of gonadotropins daily beginning on bleeding days 3–5. The GnRH antagonist protocol with administration of gonadotropins admitted on bleeding day 2/3. After 5 days of gonadotropin injection or when follicular size reaches more than 14 mm, subcutaneous administration of the GnRH antagonist (e.g., Ganirelix 0.25 mg/d subcutaneous) began. As well, in case of endometriosis, the ultra-long protocol was used correspondingly to the long protocol; however, GnRH agonist was given up to 3 months before administration of gonadotropins daily. Gonadotropins for controlled hyperstimulation were Follitropin alpha (minimal applied dosage: 100 I.E., maximally: 225 I.E.), Follitropin beta (minimal dosage: 100 I.E., maximally: 225 I.E.), Menotropin (minimally: 75 I.E., maximally: 225 I.E.) or Corifollitropin alpha depot (100 µg < 60 kg body weight or 150 µg ≥ 60 kg body weight). Gonadotropins were injected solely or in combination. Gonadotropin dosage was determined on basis of age, latest AMH and AFC, or, if existent, experience from the last treatment.

Ovulation induction (OI) was triggered with 10.000 I.E. human choriogonadotropin (hCG) injection 35 h before oocyte retrieval, if at least three follicles measured ≥ 17 mm. The duration of stimulation was determined as the time between the first dose of gonadotropin stimulation and the day of OI. Serum estradiol and progesterone levels were determined upon OI. After ovum pick-up, the total number of collected oocytes, of mature metaphase-II oocytes and of fertilized oocytes 16–19 h after IVF/ICSI procedure were recorded. Data are illustrated in Table 4.

**Table 4:** Data of follicle stimulation and assisted reproductive technologies (ART)

	Mean ± STD (range)
Timespan of follicle stimulation (days)	11.64 ± 4.04 8–16
Cycle number *n*	0 ± 1.0 0–7
Estradiol serum concentration on day of OI pg/ml	1898 ± 997 554–5676
Progesterone serum concentration on day of OI ng/ml	0.7 ± 0.6 0.1–2.7
Collected oocytes Number *n*	10.62 ± 6.23 1–28
MII oocytes Number *n*	8.5 ± 5.1 1–24
Number of fertilized oocytes	5.63 ± 3.57 1–18
Pregnancy rate (%)	35.1 ± 4.6

Collected oocytes: number of collected oocytes by oocyte retrieval, MII oocytes: number of mature metaphase-II oocytes

*OI* day of ovulation induction

The pregnancy test was assumed positive with serum hCG levels of > 5 U/mL 14 days after oocyte retrieval or the ultrasonographic report of an intrauterine gestational sac.

Data and measurements were evaluated in 3-month quartiles (1st August–31st October, 1st November–31st January, 1st February–30th April, 1st May–31st July) and impact on ART outcome was assessed.

### Statistics

Statistical analyses were performed with the Statistical Package for Social Sciences (SPSS for Windows 22.0, SPSS Inc., Chicago IL, USA). The chi-square test was used for categorical variables. Kruskal–Wallis testing was used for continuous variables and comparing more than two groups. Spearman correlation coefficient *ρ* was performed to evaluate the correlation between the variables. Evaluating the relationship of vitamin D and clinical outcomes of IVF, univariate and multivariate logistic regression analyses were utilized. A *p* value of < 0.05 was considered to be statistically significant.

## Results

### Biographic data of the patients

Biographic data of patients as well as D3 serum concentrations, AMH, AFC and BMI were measured and recorded on the day of starting controlled gonadotropin hyperstimulation for ART, respectively, days 3–5 of the bleeding. Mean values are illustrated in Table 3. Data of stimulation including ART outcome are shown in Table 4.

### Vitamin D dynamic

**Fig. 1:**
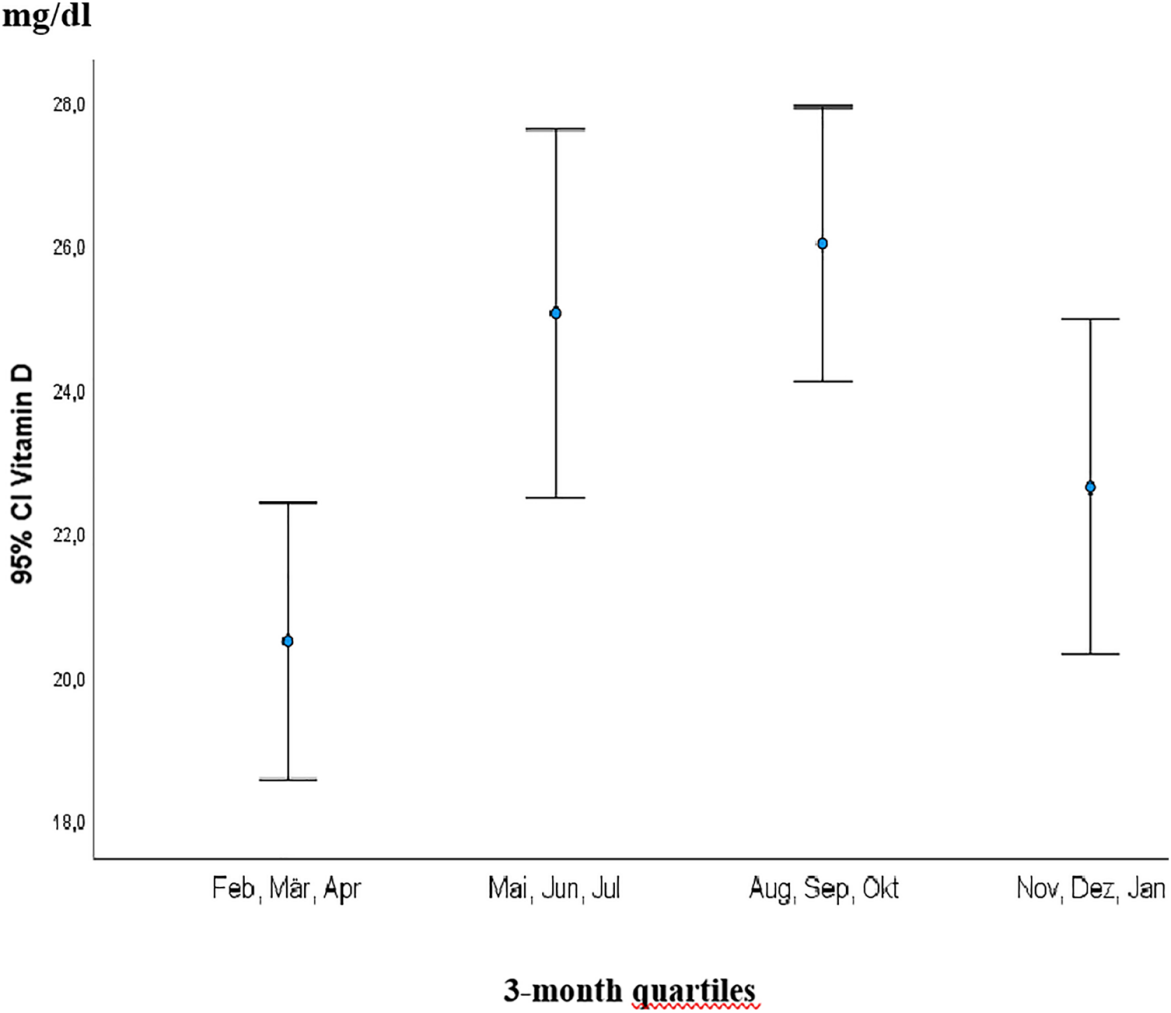
Illustration of vitamin D3 serum concentrations (mg/dl) in 3-month quartiles: highest mean serum concentration was recorded from August to October and lowest from February to April (26.0 vs. 20.5 mg/dl; *p* < 0.0001)

We could confirm and amend the results of our pilot study by Rogenhofer et al. [36] showing statistically significant 3-month quartile changes of D3 serum concentrations (*p* < 0.0001) registering the maximum level from 1st August to 31st October with a mean of 26.0 mg/dl. The lowest expression was noted from 1st February to 30th April with a mean of 20.5 mg/dl (Fig. 1).

### AMH dynamic and impact on ART

**Fig. 2:**
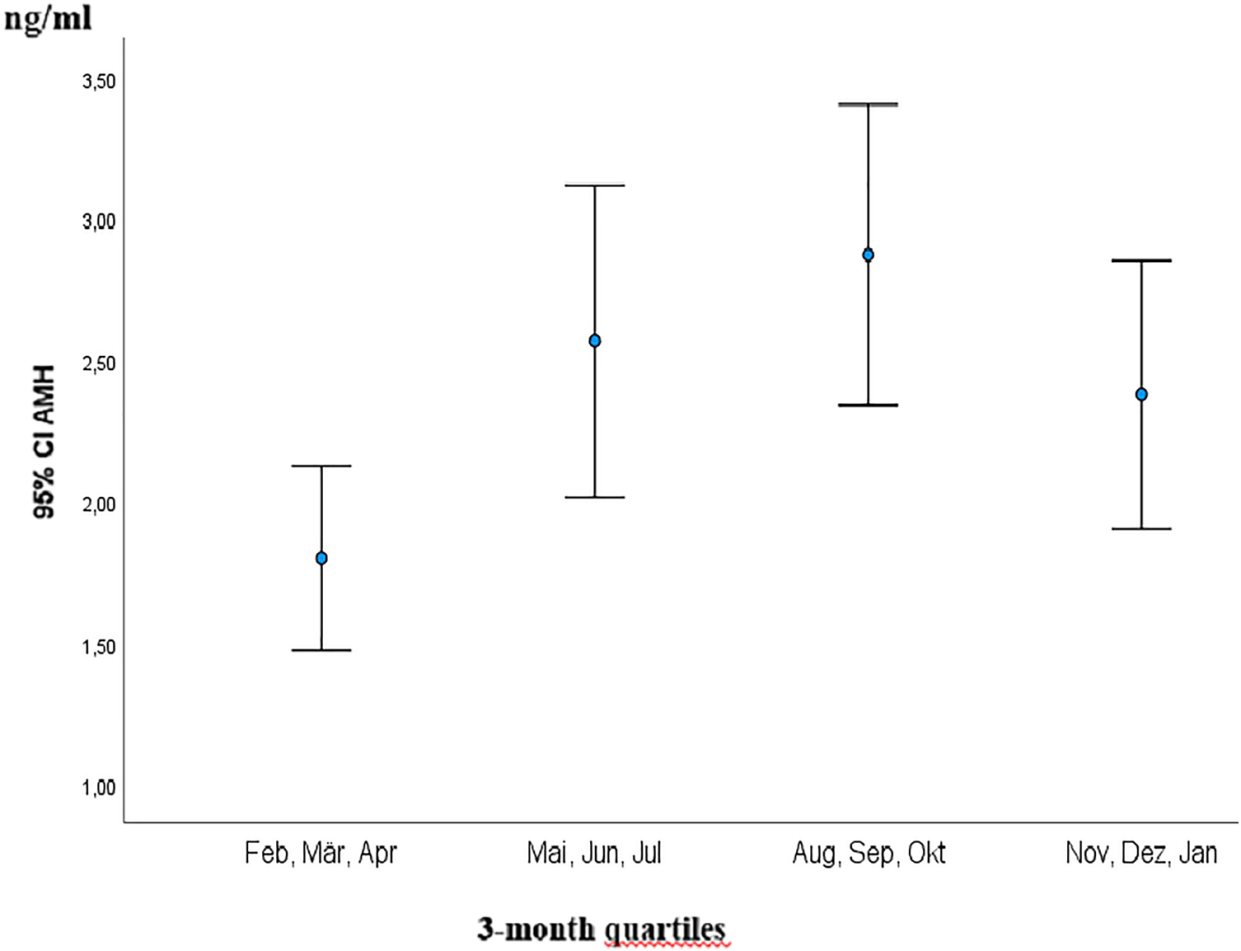
Illustration of AMH serum concentrations (ng/ml) in 3-month quartiles: highest mean serum concentration was recorded from August to October and lowest from February to April (2.98 vs. 1.78 ng/ml; *p* = 0.010) *AMH* Anti-Muellerian Hormone

**Fig. 3:**
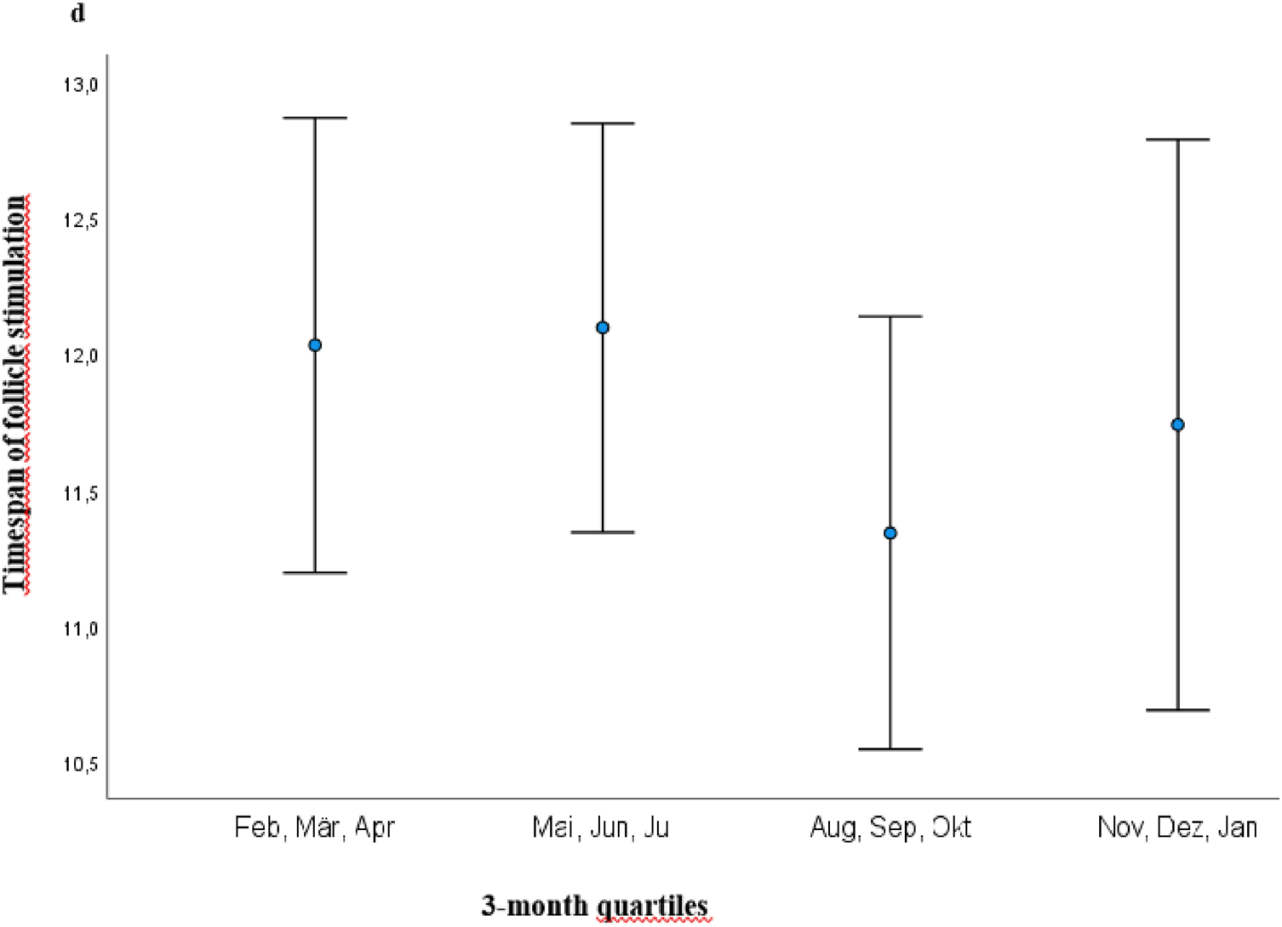
Mean timespan (in days) of follicle stimulation in 3-month quartiles: shortest timespan was recorded from August to October with mean of 11.29 days versus longest timespan of 12.12 days (*p* = 0.042). *d* days

**Fig. 4:**
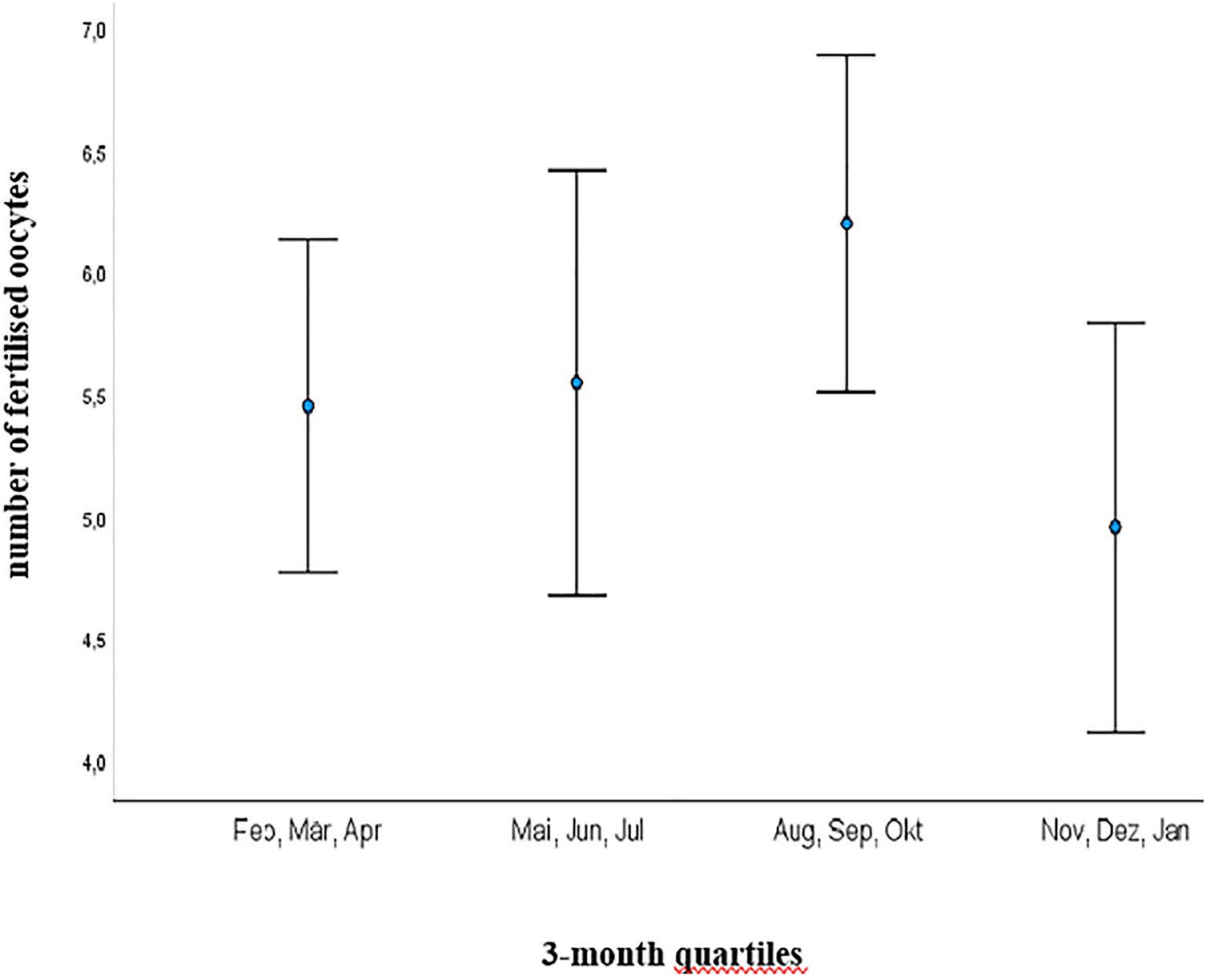
Illustration of number of fertilized oocytes in 3-month quartiles: highest number was archived from August to October with mean of 6.23 and lowest number from November to January with mean of 4.97 (*p* = 0.05)

Interestingly, AMH showed the same significant seasonal dynamic as D3 with highest concentrations from 1st August to 31st October (mean concentration of 2.98 ng/ml) and lowest from 1st February to 30th April (mean concentration of 1.78 ng/ml) (*p* = 0.010) (Fig. 2). Accordingly, the shortest time span of follicle stimulation was recorded in the quartile 1st August–31st October with a mean of 11.29 days vs. longest time span of 12.12 days (*p* = 0.042) (Fig. 3). As well, the highest number of fertilized oocytes were accomplished from August to end of October with a mean of 6.23 and lowest from November to end of January with a mean of 4.97 (*p* = 0.05) (Fig. 4).

**Fig. 5:**
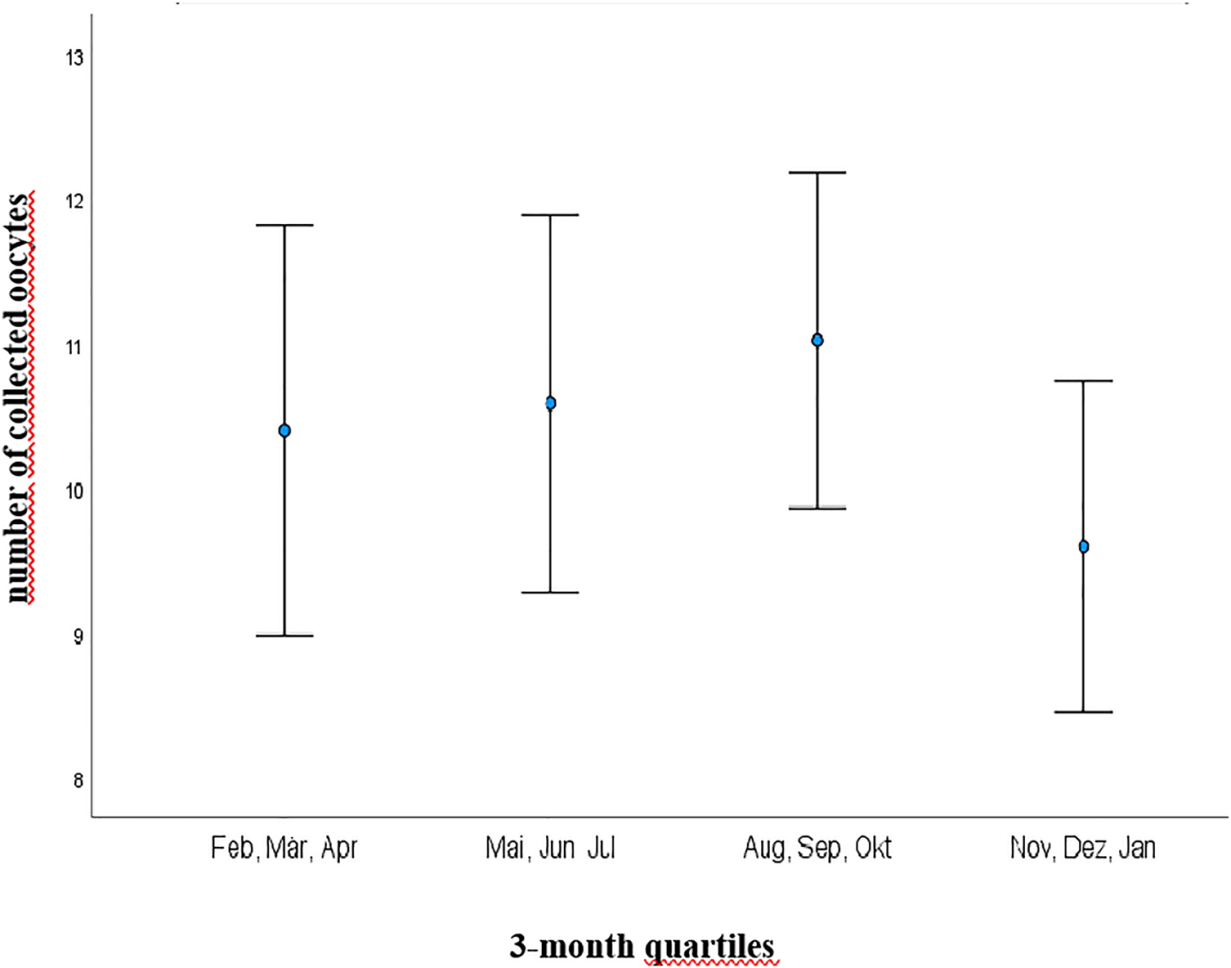
Illustration of number of collected oocytes in 3-month quartiles: highest number was collected from August to October with mean of 11.02 and lowest number from November to January with mean of 9.54 (*p* = 0.07)

A tendency to the highest number of collected oocytes was seen in the same quartile (August to end of October) with a mean number of 11.02 and minimal number with a mean of 9.54 from November to end of January (*p* = 0.07) (Fig. 5). A similar dynamic was registered for the number of metaphase-II oocytes (August–October: 9.03 vs. February–April: 8.06; *p* = 0.3), the AFC (August–October: 9.71 vs. February–April: 7.92; *p* = 0.2) as well for the pregnancy rate (August–October: 39.1% vs. May–July: 32.9%; *p* = 0.7).

## Discussion

In current literature, there are discordances if there is an association between seasonal D3 concentrations and AMH and if there is an influence on the outcome of an IVF/ICSI treatment.

In this study, we analyzed 3-month quartile D3 fluctuations and the impact on AMH and ART outcome. Our data are in line with several in vitro and in vivo studies reporting seasonal dynamic of D3 and the impact on AMH [1]. However, our findings show a parallel 3-month dynamic as AMH with peak levels of both, D3 and AMH delayed from August to end of October, supporting the hypothesis of our pilot study suggesting a prolonged tissue storage with a following slow release of D3 [36]. Here, in addition, a similar quartile dynamic was found in the ART outcome with best results from August to end of October.

Recently, a study group investigated whether 25(OH)D is associated with IVF outcome in 848 infertile Chinese women undergoing IVF, as well, grouped in seasonal quartiles, i.e., spring (February–April), summer (May–July), autumn (August–October), winter (November–January) [34]. In accordance with our results, this study found the highest 25(OH)D levels in autumn with a minimum in spring. However, AMH levels developed divergently from vitamin D levels, even a trend of worsening vitamin D status with increasing AMH was observed [34]. Nonetheless, in line with our results and in accordance with another large previous investigation [38], fertilization rates peaked in autumn from August to October. Accordingly, the seasonal quartile variations, which in turn are associated with fluctuating vitamin D levels, seem to be of importance for ART treatment. Though, the process of fertilization depends on several factors. For example, the quality of the oocyte, the adhesion and penetration of the sperm to the zona pellucida [34]. D3 might support oocyte maturation prior to oocyte retrieval, resulting in a greater capability to achieve fertilization by improving the interaction of the sperm with the zona pellucida [34].

On the other hand, some studies on populations of women with infertility, yielding negative association between serum vitamin D and AMH concentrations [1, 21, 28, 30, 31].

Instance, Drakopoulos et al. could not verify an association between seasonal vitamin D and AMH in 283 patients undergoing fertility treatment [31]. To note, the authors did not take the ethnicity into account, although women with dark skin were identified to have a more likely vitamin D deficiency [21]. In addition, a prospective trial with 656 participants grouped in two semesters (winter/spring and summer/autumn) found no association between 25(OH)D concentrations and AMH, respectively, the risk of early menopause [28].

Last but not least, the most recent review and meta-analysis of 18 observational and 6 interventional studies, evaluating the relationship between serum vitamin D and AMH levels, report rather discrepant results [1].

Several reasons may account for the conflicting literature: different race/ethnicity, geographic location, seasonality and the heterogeneity of the populations. Some studies looked at normal ovulatory women, while others looked at women with PCOS or with diminished ovarian reserve or vitamin D deficient and nondeficient women. However, only a few of the numerous studies take the seasonal fluctuations of vitamin D into account, even though this phenomenon is widely known.

Due to this fact, we performed seasonal classification in 3 monthly quartiles, not according to calendrical quarters, but with a delay of 1 months, i.e., August–October, November–January, February–April, May–July, with regard to our pilot study postulating a prolonged tissue storage with a following slow release of D3 resulting in a delay of the D3 deficiency [36].

Further assets that strengthen our work are as follows: our study includes a relatively large sample size of 469 women undergoing IVF/ICSI with strict inclusion criteria. AMH and D3 were analyzed at the same time without storing the blood samples, reflecting the actual status of both hormones. Moreover, all AMH levels were measured in the same laboratory by the same test method, i.e., Electrochemiluminescence immunoassay (ECLIA) (Elecsys Cobas, Roche Diagnostics International Ltd.). This fact is of importance, because this assay is a fully automated platform approved for a stable intra- and inter-assay coefficient of variation and highly functional sensitivity, especially since the stability of AMH and the high sample-to-sample variability under different assays has been criticized [39]. Additionally, the group of included patients was rather homogeneous: European descent mainly living in Germany; no participant was dark-skinned, as women with dark skin tone seem to have more likely a vitamin D deficiency [21], tend to have lower serum AMH levels (16) and an earlier onset of menopause [28, 40–42]. In addition, we excluded women with PCOS to avoid interactions of D3 with PCOS, as recent studies showed that PCOS patients reveal increased AMH levels [1, 12]. Moreover, we avoided any kind of vitamin D supplementation, considering data of improving hormonal disorders by vitamin D intake [1, 13, 28, 40–42].

However, to discuss possible limitations of our study: we did not measure D3 and AMH in the same person in each quartile over the year. These data would clearly confirm indication for a seasonal impact. Nevertheless, our findings assume a parallel 3-month dynamic of D3 and AMH—probably based on the fact of the existence of the vitamin D response element in the AMH gene [19, 20]—with peak delayed to autumn month and similar quartile dynamic in the ART outcome.

Further, to note critically, that AMH is a stable prognostic marker for the amount of oocytes that might be retrieved after stimulation in an IVF/ICSI cycle, but it is a poor marker for predicting a pregnancy [43], as it is only weakly associated with embryo implantation [17]. The majority of studies suggested no statistically association between clinical pregnancy rates or life birth rates [1, 4, 6, 30, 34, 44], probably due to specific framework of the studies. However, Brodin et al. showed a strong association between AMH and live birth rates after ART [45]. These findings may indicate a relation between AMH, vitamin D and oocyte quality. As we did not focus on live birth rates in this study, it is our goal for future work.

## Conclusion

In conclusion, our results show that AMH is subjected to seasonal 3-monthly fluctuations with a peak in autumn from August to end of October. Interestingly, our data also suggest a positive correlation of the IVF/ICSI outcome in terms of fertilization rate and the total number of collected oocytes with D3 serum concentrations, thus rendering the D3 status an additional focal point of attention at the beginning of fertility treatments.
